# Circ_0088194 Promotes the Invasion and Migration of Rheumatoid Arthritis Fibroblast-Like Synoviocytes *via* the miR-766-3p/MMP2 Axis

**DOI:** 10.3389/fimmu.2021.628654

**Published:** 2021-02-22

**Authors:** Yujie Cai, Renge Liang, Shibai Xiao, Qin Huang, Dingji Zhu, Guo-Ping Shi, Qingqing Ouyang, Min Yang

**Affiliations:** ^1^ Department of Rheumatology and Immunology, Nanfang Hospital, Southern Medical University, Guangzhou, China; ^2^ Department of Medicine, Brigham and Women’s Hospital and Harvard Medical School, Boston, MA, United States

**Keywords:** rheumatoid arthritis, fibroblast-like synoviocytes, CircRNA, Circ_0088194, miR-766-3p, matrix metalloproteinase 2

## Abstract

Dysregulation of circular RNAs (circRNAs) is involved in various human diseases. Fibroblast-like synoviocytes (FLSs), which form the lining of the joint, are epigenetically imprinted with an aggressive phenotype and contribute to joint destruction in rheumatoid arthritis (RA). In the present study, we identified a novel circRNA, Circ_0088194, which was upregulated in RA fibroblast-like synoviocytes (RA-FLSs) and correlated with the disease activity score in 28 joints. Overexpression of Circ_0088194 promoted RA-FLS migration and invasion, while inhibition of Circ_0088194 had the opposite effect. Mechanistically, Circ_0088194 acted as a miR-766-3p sponge to relieve the repressive effect of miR-766-3p on its target, *MMP2* (encoding matrix metalloproteinase 2), thereby promoting migration and invasion. The expression level of Circ_0088194 was inversely correlated with that of miR-766-3p in RA-FLSs. Importantly, overexpression of miR-766-3p partially blocked the migration and invasion induced by Circ_0088194 overexpression. Collectively, this study identified a novel circRNA Circ_0088194 that promotes RA-FLS invasion and migration *via* the miR-766-3p/MMP2 axis. Circ_0088194 might represent a novel therapeutic target to prevent and treat RA.

## Introduction

Rheumatoid arthritis (RA) is one of the most prevalent chronic inflammatory diseases, and is characterized by progressive destruction of cartilage and bone, with concomitant disability (1). This disease carries a substantial burden for both the affected individuals and for society (2). Recently, the treatment of RA has improved, such as the use of combination anti-rheumatic drug strategies that modify the disease and the development of biological therapies; however, overall remission rates are still unsatisfactory (3). Therefore, it is imperative to determine the molecular mechanisms responsible for the development and progression of RA, and to identify new target molecules for early treatment.

In the formation of invasive synovial tissue, the key cells are RA fibroblast-like synoviocytes (RA-FLSs), which play a major role in the RA pathological processes (4). In RA pathogenesis, activated RA-FLSs exhibit similar characteristics to tumor cells, such as enhanced migration and invasion abilities, which promote RA progression, ultimately leading to joint destruction (5). Thus, investigating the mechanisms responsible for RA-FLs invasion and migration might identify potential therapeutic strategies to treat RA.

RNA transcripts forming covalently closed loops produced by direct non-canonical back-splicing are known as circular RNAs (circRNAs) (6, 7). CircRNAs are very stable and are highly abundant in various tissues because of their circular structure (8). CircRNA sequences are highly conserved and circRNAs are expressed in a tissue or cell-specific manner (9, 10). Recent studies indicated that circRNAs have diverse biological functions and are associated with the pathogenesis of various diseases, including cancer (11), cardiovascular disease (12), neurological disorders (13), and osteoarthritis (OA) (14). Importantly, the regulation of human cancer cell migration and invasion involves several circRNAs. For example, in non-small cell lung cancer, migration and invasion are promoted by the circRNA F-circEA-2a, which is expressed from the *EML4-ALK* fusion gene (15); and circRNA circNHSL1 promotes gastric cancer cell migration and invasion through the miR-1306-3p/SIX1/Vimentin axis (16). These reports suggested that circRNAs play an important role in the regulation of migration and invasion. However, the roles of circRNAs in RA-FLSs are largely unknown.

The most abundant internal modification of mRNAs and long non-coding RNAs is N6-methyladenosine (m^6^A) (17, 18), which plays vital roles in stabilizing circRNAs and mRNAs (19, 20), in alternative splicing (21), and in miRNA (microRNA) biogenesis (22). Furthermore, m^6^A methylation is found in various physiological and pathological processes, such as neurogenesis (23) and cancer (24). However, whether the N6-methyladenosine modification affects the stability of circRNAs in RA-FLSs remains unknown.

In the present study, a novel circRNA, Circ_0088194, was identified in human RA-FLSs that was associated positively with RA disease activity and was responsible for RA-FLS migration and invasion by binding to miR-766-3p, thus promoting matrix metalloproteinase 2 *(MMP2)* expression.

## Materials and Methods

### Patients and Specimens

Osteoarthritis (OA), a degenerative disease with generally less severe clinical symptoms and pathology, has been utilized as a control in studies of RA pathology. From January 2017 to December 2017, nine RA synovial tissues and seven OA synovial tissues were acquired from patients with end-stage symptomatic hip RA or OA at the time of total hip replacement surgery, as performed at the Department of Orthopedic Surgery, Nanfang Hospital, Southern Medical University, Guangzhou, China. The Ethics Committee of the Southern Medical University approved the study and its associated protocols (Guangzhou, China, NFEC-20120201). All participants satisfied the criteria from the American College of Rheumatology to classify RA (25) or OA (26). Prior to enrollment, each participant provided written informed consent. The participants’ clinical parameters are shown in **Table 1**.

**Table 1:** Clinical characters and laboratory measures of the participants.

	RA (n = 9)	OA (n = 7)
Age (year, mean ± SD)	51.11 ± 2.72	51.71 ± 2.10
Gender (male/female)	2/7	2/5
Anti-CCP* (U/ml)	37.59 (7.80–1,121.50)	NA*
DAS28* (scores)	7.01 (3.08–7.71)	NA*

*DAS28, disease activity score in 28 joints; anti-CCP, anti-cyclic citrullinated peptide antibodies; NA, not available.

### Fibroblast-Like Synoviocytes (FLSs) Culture

FLSs were isolated from human synovial tissue specimens and cultured in Dulbecco’s modified Eagle’s medium (DMEM) (Thermo Fisher Scientific, Inc., Waltham, MA, USA) with 10% added fetal bovine serum (FBS) (Gibco BRL, Grand Island, NY, USA). FLSs were passaged three to five times before being used and the RA-FLSs were confirmed using vimentin immunofluorescent staining as a homogenous population with purity >98% (**Supplementary Figure 1**).

### CircRNA Microarray Analysis

Three different samples of RA-FLSs and OA-FLSs were used for microarray analysis, which was performed by Kangcheng Bio-tech Inc. (Shanghai, China). Briefly, RNase R (Epicentre Inc., Madison, WI, USA) was used to digest the total RNA, which removed linear RNAs, thus enriching circRNAs. An Arraystar Super RNA Labeling Kit (Arraystar Inc., Rockville, MD, USA), together with the random-priming method, was used to amplify and transcribe the enriched circRNAs into fluorescent circRNAs. Complementary DNA (cDNA) was synthesized and labeled before array hybridization. Then, the fluorescent circRNAs were hybridized to the Arraystar Human circRNA Microarray V2.0 (8 × 15K, Arraystar Inc.). Thereafter, the slides were washed and an Agilent Scanner G2505C (Agilent Technologies, Santa Clara, CA, USA) was used to scan the arrays. Analysis of the acquired array images was carried out using Agilent Feature Extraction software (version 11.0.1.1). The R software package (R version 3.1.2) was used for quantile normalization and subsequent data processing.

### Quantitative Real-Time Polymerase Chain Reaction (qRT-PCR)

Total RNA was obtained from cultured cells using the TRIzol reagent (Takara, Shiga, Japan), according to the manufacturer’s instructions, and reverse transcribed into cDNA. The qPCR step was carried out using the cDNA as the template and SYBR Premix DimerEraser (Takara) with the ABI7500 system (Applied Biosystems, Foster City, CA, USA). Relative expression was calculated using the relative quantification (2−ΔΔCt) method (27). *GAPDH* (encoding glyceraldehyde-3-phosphate dehydrogenase) was used as an internal control for circRNAs and mRNAs, and U6 was employed as an endogenous control for the miRNAs. **Supplementary Table S1** shows the details of the primers used for qRT-PCR.

### Electrophoresis of Nucleic Acids

Agarose gel electrophoresis (4%) with Tris acetate-ethylenediaminetetraacetic acid running buffer (Thermo Fisher Scientific) was used to analyze genomic DNA (gDNA), PCR products, and cDNAs. Electrophoresis was performed at 110 V for 50 min to isolate DNA. A 20 bp DNA marker (Takara) was used and bands were examined by ultraviolet irradiation.

### Fluorescence *In Situ* Hybridization (FISH)

FISH analysis of RA-FLSs used biotin-labeled probes specific to Circ_0088194 (GenePharma Co. Ltd., Shanghai, China). FISH (GenePharma) was used to detect the signals of these probes according to the manufacturer’s instructions. 4, 6-diamidino-2-phenylindole (DAPI) was used to counterstain the nuclei. A Leica TCS SP2 AOBS confocal microscope (Leica Microsystems, Mannheim, Germany) was used to acquire images. The probe sequences are listed in **Supplementary Table S1**.

### Western Blotting Analysis

Cultured RA-FLSs were lysed in ice-cold radioimmunoprecipitation assay buffer (BestBio, Shanghai, China) containing phosphatase inhibitors and a protease inhibitor cocktail (Sigma-Aldrich, St. Louis, MO, USA). The proteins in the cell lysates were separated using 10% SDS-PAGE, followed by electroblotting onto a polyvinylidene difluoride membrane (Millipore, Billerica, MA, USA). Incubation for 1 h at room temperature in Tris-buffered saline with Tween-20 and 5% skim milk was used to block the membrane. The membrane was then probed using primary antibodies recognizing rabbit matrix metalloproteinase 2 (MMP2) (1:5,000; Bioworld, Bloomington, MN, USA) and GAPDH (1:10,000; Bioworld) overnight at 4°C. Next day, the blot was incubated with horseradish peroxidase-conjugated secondary antibodies (1:10,000; Fdbio science, Hangzhou, China). The signals from the immunoreactive proteins were quantified using the Quantity One Software (Bio-Rad, Hercules, CA, USA).

### Adenoviral Construction and Transduction

The adenoviral expression vectors for Circ_0088194 were constructed by Genepharma (Shanghai, China). To overexpress Circ_0088194, RA-FLSs were transduced with an adenoviral expression vector expressing Circ_0088194 or an empty adenoviral vector control following the manufacturer’s protocol. QRT-PCR was used to determine the Circ_0088194 expression levels.

### Oligonucleotides and siRNA Transfection

Small interfering RNAs (siRNAs), miRNA mimics, and miRNA inhibitors were obtained from the RiboBio (Guangzhou, China). Cells were transfected with 50 nM of Circ_0088194 siRNAs, *MMP2* siRNAs, miR-766-3p mimics, miR-766-3p inhibitors, or the corresponding controls using RNAiMAX (RiboBio) according to the manufacturer’s instructions. All relevant sequences are listed in **Supplementary Table S1**.

### Assays for Cell Migration and Invasion

For the migration assay, cells [1×10^4^ in 200 μl of DMEM (serum free)] were seeded onto the top chamber of a Transwell insert, then DMEM with 10% FBS (600 μl) was added to the bottom chamber. The invasion assay started the same, except that in addition to the above, 50 µl of Matrigel (BD Biosciences, Franklin Lakes, NJ, USA) was layered onto the top chamber. The chambers were then incubated for 48 h. Thereafter, a cotton swab was used to remove the cells remaining on the on the surface of the upper membrane surface. Crystal violet was used to stain the cells that had crossed the membrane. Under a microscope, cells in six random fields were counted. These assays were carried out three times independently.

### RNA Immunoprecipitation (RIP)

RIP experiments were conducted following the manufacturer’s instructions of the Magna RIP RNA-Binding Protein Immunoprecipitation Kit (Millipore, Bedford, MA, USA). Briefly, MiR-766-3p mimics or the negative control were transfected into RA-FLSs. After 48 h, the RA-FLSs were lysed using RIPA buffer (Cell Signaling Technology, Danvers, MA, USA). Magnetic beads (Invitrogen, Waltham, MA, USA) were pre-incubated with anti-AGO2 antibodies or anti-rabbit IgG (Cell Signaling Technology) for 30 min, and then the lysates were immunoprecipitated using the beads, with rotation overnight at 4°C. Next day, the RNA was purified from the RNA–protein complexes bound to the beads, and then the levels of Circ_0088194 and miR-766-3p were determined using qRT-PCR followed by agarose gel electrophoresis.

### Protein Array Analysis

RA-FLS cell lysate protein samples were assayed using a Human Cytokine Array GS440 (Cat#GSH-CAA-440, RayBiotech, Inc., Norcross, GA, USA) and processed according to the manufacturer’s instructions. Briefly, the array slide was incubated with 65 µl of RA-FLS cell lysate overnight at 4°C. The glass array slide was equilibrated to room temperature on the following day, and washed extensively before incubating with an array-specific biotinylated antibody cocktail for 2 h. Unbound antibody was washed away, and the slide was developed for 1 h with Cy3-equivalent dye-conjugated streptavidin. Images were captured using an InnoScan 300 Microarray Scanner (Innopsys, Chicago, IL, USA), and positive clones were identified using the ScanArray Express analysis software (PerkinElmer, Boston, MA, USA). To minimize false positive hits, each sample was screened twice and only the hits that appeared in both screens were analyzed. In this manner, a unique antibody profile was generated for each sample. Experiments were carried out in duplicate.

### Bioinformatic Analysis

Potential miRNA targets were predicted using three publicly available databases: TargetScan, miRanda (http://www.microrna.org/microrna/home.do), and circular RNA interactome (https://circinteractome.nia.nih.gov). Targets were accepted only when they appeared in all three databases.

### Dual-Luciferase Reporter Assay

The Circ_0088194 segment and a fragment of *MMP2* mRNA were synthesized from either mutant (MUT) or wild-type (WT) seed regions and cloned into the psiCHECK-2 vector (Promega, Madison, WI, USA). To generate mutant Circ_0088194 sequences, 11 nucleotides of the seed region were changed (WT seed sequence: 5′-CTGT … GCTGGAG-3′, MUT seed sequence: 5′-GACA … CGACCTC-3′). To generate the mutant *MMP2* mRNA sequence, we mutated five nucleotides of the seed region (WT seed sequence: 5′-CTGGA-3′; MUT seed sequences: 5′-GACCT-3′). RA-FLSs (1 × 10^5^) were transfected with either WT or MUT Circ_0088194, miR-766-3p mimics or mimics control, and WT or MUT *MMP2* mRNA, using Lipofectamine 2000 (Thermo Fisher Scientific). After induction for 48 h, luciferase activity was assessed using a dual-luciferase reporter kit (Promega).

### Measurement of m^6^A-modified Circ_0088194

Quantification of m^6^A-modified Circ_0088194 was achieved using methylated RNA immunoprecipitation. An aliquot (1.5 μg) of anti-m^6^A antibody (Santa Cruz Biotechnology, Santa Cruz, CA, USA) or anti-IgG antibody (Santa Cruz Biotechnology) was incubated with protein A/G magnetic beads overnight at 4°C. An aliquot (100 ng) of total RNA and m^6^A spike-in control mixture were added to 300 μl of an IP buffer (50 mM Tris-HCl, pH 7.4, 150 mM NaCl, 0.1% NP40, 40 U/μl RNase Inhibitor) containing 2 μg of anti-m^6^A rabbit polyclonal antibody. RNA was eluted using elution buffer, purified using phenol-chloroform extraction, and subjected to qRT-PCR analysis.

### Immunofluorescent Staining

RA-FLSs were fixed for 20 min using 4% paraformaldehyde, and then permeabilized for 5 min at room temperature using 0.2% Triton X-100. The cells were then washed and blocked for 1 h at room temperature using 10% normal goat serum (BioSS, Beijing, China), followed by incubation at 4°C overnight with antibodies recognizing vimentin (1:100). Next day, the cells were washed with phosphate buffered saline three times, followed by incubation for 1 h at room temperature with goat anti-rabbit IgG/Alexa Fluor 647 antibodies (1:200). Nuclei were counterstained with DAPI and images were acquired on a Leica TCS SP2 AOBS confocal microscope (Leica Microsystems).

### Statistical Analysis

All experiments were carried out three times. Data are shown as the mean ± the standard deviation (SD). For comparisons between two groups or among multiple groups, we used Student’s t-test and one-way analysis of variance (ANOVA), respectively. Spearman rank correlation was used to analyze the association of Circ_0088194 expression with disease activity. All statistical analyses were performed using the SPSS 20.0 software (IBM Corp. Armonk, NY, USA). *P <*0.05 was considered statistically significant.

## Results

### Expression Profile of Circular RNAs in OA-FLSs and RA-FLSs

**Figure 1:**
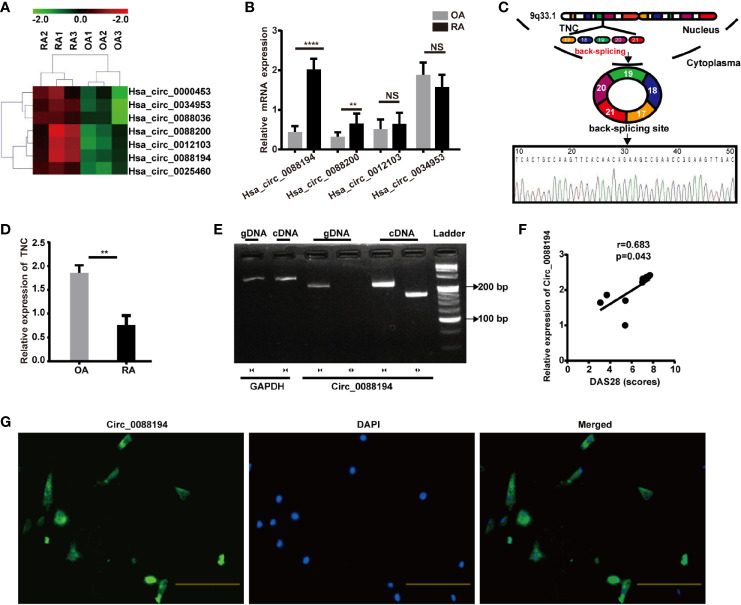
Circ_0088194 is upregulated in RA-FLSs. **(A)** Hierarchical clustering analysis of differentially expressed CircRNAs between RA-FLSs and OA-FLSs. **(B)** QRT-PCR was used to detect the circRNA expression levels in nine RA-FLSs and seven OA-FLSs. (RA-FLSs = 9, OA-FLSs = 7). **(C)** Schematic illustration of how the Circ_0088194 is back-spliced from tenascin C (*TNC*) exons 17 to 21. The existence of Circ_0088194 was validated using qRT-PCR, followed by Sanger sequencing. **(D)** The *TNC* mRNA expression level, as detected using qRT-PCR in nine RA-FLSs and seven OA-FLSs. (RA-FLSs = 9, OA-FLSs = 7). **(E)** Divergent primers could amplify Circ_0088194 from cDNA but not from gDNA. **(F)** Circ_0088194 expression in RA-FLSs correlated positively with the RA disease activity score in 28 joints (DAS28 score). (n = 9; r = 0.683, *P* = 0.043). **(G)** Circ_0088194 showed a predominantly cytoplasmic localization upon RNA FISH. Scale bar, 500 µm. Data are shown as mean ± SD. NS, not significant. **indicates *p <* 0.01, and ****indicates *p <* 0.0001.

FLSs samples from three patients with RA and samples from three age- and sex-matched patients with OA were subjected to microarray analysis of circRNAs to assess their expression (**Supplementary Figure S2**). Hierarchical clustering showed variations in circRNA expression between RA-FLSs and OA-FLSs (**Figure 1A**). Seven circRNAs were identified as being differentially expressed (fold change > 2.5 and *p* < 0.05) between RA-FLSs and OA-FLSs (**Table 2**). Next, we selected exonic circRNAs that exhibited >3-fold change (*p* < 0.05) and detected their expression levels in nine RA-FLSs and seven OA-FLSs to verify the microarray results. The results showed that the expression levels of Circ_0088194 and Circ_0088200 were elevated in RA-FLSs compared with those in OA-FLSs, while the expression levels of Circ_0012103 and Circ_0034953 did not differ between the two cell populations (**Figure 1B**). These differentially expressed circRNAs between RA-FLSs and OA-FLSs may have potential functions in RA progression.

**Table 2:** Microarray analysis of dysregulated circRNAs in three RA patients compared with three OA patients.

CircRNA	P-value	Fold change	Regulation	CircRNA type	Gene Symbol
Hsa_circ_0088200	0.027778	3.503261	up	exonic	*TNC*
Hsa_circ_0012103	0.018074	3.401054	up	exonic	*ST3GAL3*
Hsa_circ_0034953	0.023194	3.202578	up	exonic	*TUBGCP4*
Hsa_circ_0088194	0.015054	3.095609	up	exonic	*TNC*
Hsa_circ_0000453	0.03798	2.733945	up	antisense	*BCL7A*
Hsa_circ_0088036	0.048633	2.673338	up	exonic	*SUSD1*
Hsa_circ_0025460	0.004475	2.601944	up	exonic	*YBX3*

### Circ_0088194 Is Highly Expressed in the Cytoplasm of RA-FLSs and Correlated With the Disease Activity Score in 28 Joints

The expression levels of Circ_0088194 were the highest among the differentially expressed circRNAs in RA-FLSs compared with that in OA-FLSs (**Figure 1B**). Therefore, Circ_0088194 was chosen for further investigation. The gene encoding this circRNA is located at chr9:117798376–117808961 and this circRNA is formed by the reverse splicing of exons 17 to 21 of the tenascin C (*TNC*) gene (**Figure 1C**). We found that the expression levels of *TNC* were downregulated significantly in RA-FLSs compared with those in OA-FLSs (**Figure 1D**). The existence of Circ_0088194 was confirmed in numerous circRNA databases. According to the circBase database (http://circrna.org/cgi-bin/singlerecord.cgi?id=hsa_circ_0088194), Circ_0088194 exists in many tissue and cell types, such as normal human epidermal keratinocytes. We then used Sanger sequencing to confirm the head-to-tail splicing (formed from a 5′-end splice site and the corresponding 3′-end site of exons) using the Circ_0088194 qRT-PCR product, which was identified by its expected size (**Figure 1C**).

To rule out the possibility that genomic rearrangements or trans-splicing caused the head-to-tail splicing, two groups of primers were designed: Divergent primers were used to amplify Circ_0088194, and convergent primers were used to amplify the *TNC* mRNA. The cDNA and gDNA samples were used as templates for qRT-PCR. Using the divergent primers, we could amplify Circ_0088194 using the cDNA as a template, but not using the gDNA (**Figure 1E**). To assess the correlation between Circ_0088194 expression levels and RA disease activity in nine RA-FLS samples, Spearman’s rank correlation was used. We found that the Circ_0088194 levels correlated positively with the disease activity score in 28 joints (DAS28) (r = 0.683, *p* = 0.043) (**Figure 1F**). Moreover, a FISH assay revealed that Circ_0088194 was localized mostly in the cytoplasm of RA-FLSs, but not in the nucleus (**Figure 1G**). Thus, Circ_0088194 is associated with RA disease severity and is highly expressed in the cytoplasm of RA-FLSs.

### Circ_0088194 Promotes RA-FLS Migration and Invasion

**Figure 2:**
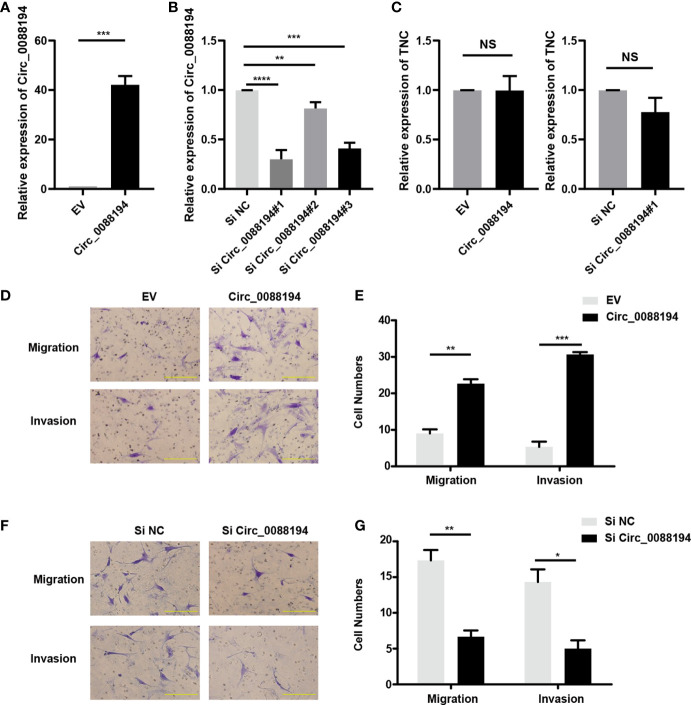
Circ_0088194 promotes RA-FLS migration and invasion. **(A)** RA-FLSs were infected with adenovirus expressing Circ_0088194 or with the empty vector (EV). The expression levels of Circ_0088194 were detected using qRT-PCR. **(B)** RA-FLSs were transfected with three Circ_0088194 siRNAs or the negative control (NC). The Circ_0088194 expression level was measured using qRT-PCR **(C)** RA-FLSs were infected with adenovirus expressing Circ_0088194 or Circ_0088194#1 siRNA, respectively. The Tenascin C (*TNC*) expression level was measured using qRT-PCR. **(D, E)** Transwell migration and Matrigel invasion assays were used to evaluate the migratory and invasive activity of RA-FLSs. RA-FLSs were infected with adenovirus expressing Circ_0088194 or the EV. Scale bar, 500 µm. **(F, G)** RA-FLSs transfected with Circ_0088194#1 siRNA, or NC. Transwell migration and Matrigel invasion assays were used to evaluate the migratory and invasive activity of RA-FLSs. Scale bar, 500 µm. Data are shown as mean ± SD. NS, not significant. *indicates *p* < 0.05, **indicates *p <* 0.01, ***indicates *p <* 0.001, and ****indicates *p <* 0.0001.

To evaluate the functions of Circ_0088194 in human RA-FLSs, an adenoviral vector encoding Circ_0088194 was constructed and three siRNAs targeting the junction sites of Circ_0088194 were designed. Circ_0088194 expression in RA-FLSs increased significantly after transduction with adenoviral vector expressing Circ_008819 (**Figure 2A**). The expression levels of Circ_0088194 decreased significantly after transfection with siRNAs targeting Circ_0088194 (**Figure 2B**). Interestingly, overexpression or knockdown of Circ_0088194 did not affect *TNC* mRNA levels (**Figure 2C**). However, overexpression of Circ_0088194 significantly promoted the migration and invasion of RA-FLSs (**Figures 2D, E**). Conversely, knockdown of Circ_0088194 significantly inhibited RA-FLS migration and invasion (**Figures 2F, G**). Taken together, these results indicated that Circ_0088194 promotes the migration and invasion of RA-FLSs.

### Circ_0088194 Regulates MMP2 Expression in RA-FLSs

**Figure 3:**
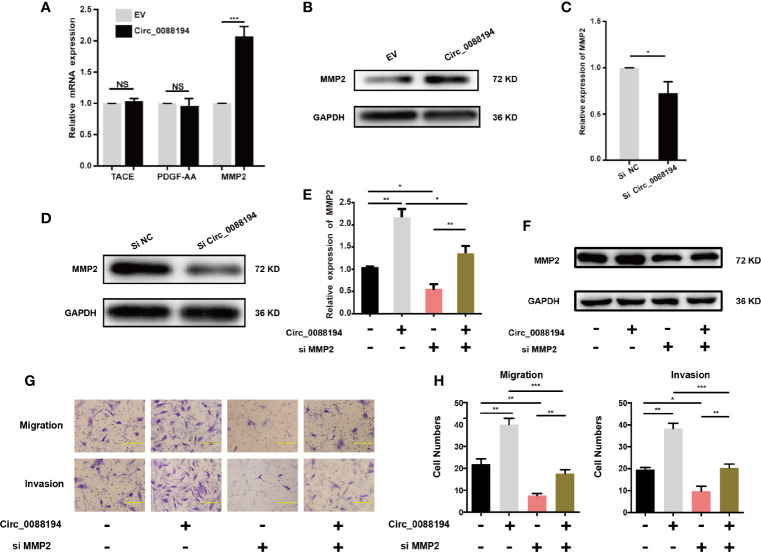
MMP2 expression in RA-FLSs is regulated by Circ_0088194. **(A)** Adenovirus expressing Circ_0088194 or empty vector (EV) were transfected into RA-FLSs. QRT-PCR determined tumor necrosis factor-α converting enzyme (*TACE*), platelet derived growth factor subunit AA (*PDGF*-*AA*), and matrix metalloproteinase 2 (*MMP2*) mRNA levels. **(B)** Adenovirus expressing Circ_0088194 or EV were transfected into RA-FLSs. Western blotting was used to detect MMP2 expression. **(C, D)** The *MMP2* mRNA and protein expression levels were examined in RA-FLSs transfected with Circ_0088194#1 siRNAs or the negative control (NC). **(E, F)** Western blotting and qRT-PCR detection of *MMP2* expression. RA-FLSs were co-transfected with the adenovirus expressing Circ_0088194 or MMP2 siRNA. **(G, H)** Transwell migration and Matrigel invasion assays were used to evaluate the migratory and invasive activity of RA-FLSs. Adenovirus expressing Circ_0088194 or MMP2 siRNA were co-transfected into RA-FLSs. Scale bar, 500 µm. Data are shown as mean ± SD. NS, not significant. *indicates *p* < 0.05, **indicates *p <* 0.01, and ***indicates *p <* 0.001.

To investigate how Circ_0088194 promotes RA-FLS invasion, we performed a protein array analysis in RA-FLSs that overexpressed Circ_0088194. The protein array analysis revealed that the levels of nine proteins were downregulated, while four proteins were upregulated in cells overexpressing Circ_0088194 (fold change > 3, *p* < 0.05) (**Table 3**). Furthermore, qRT-PCR was used to analyze the mRNA expression of three upregulated proteins (fold change > 4 and *p* < 0.05): Tumor necrosis factor-α (TNF-α) converting enzyme (TACE), matrix metalloproteinase 2 (MMP2), and platelet derived growth factor subunit AA (PDGF-AA), all of which might promote RA-FLS invasion. The results showed that only MMP2 levels, and not TACE or PDGF-AA, levels, increased in RA-FLSs after Circ_0088194 overexpression (**Figure 3A**). Moreover, western blotting analysis confirmed the elevated MMP2 levels in Circ_0088194-overexpressing RA-FLSs (**Figure 3B**). In addition, western blotting and qRT-PCR showed that knockdown of Circ_0088194 reduced the expression of MMP2 (**Figures 3C, D**). MMP2 is one of the most important proteases that mediate fibrillar collagen degradation. Several studies have indicated that MMP2 promotes the migration and invasion of glioma cells (28), lung cancer cells (29), and RA-FLSs (30, 31). Thus, we sought to determine whether Circ_0088194 promotes RA-FLSs migration and invasion by increasing MMP2 expression. Downregulation of MMP2 decreased the expression level of MMP2 induced by overexpression of Circ_0088194 (**Figures 3E, F**). Consistently, we found that inhibition of MMP2 in RA-FLSs blocked Circ_0088194 overexpression-induced migration and invasion (**Figures 3G, H**). Taken together, these results showed that Circ_0088194 promotes the migration and invasion of RA-FLSs at least partly dependent on MMP2.

**Table 3:** Protein array analysis of dysregulated proteins in three overexpressing Circ_0088194 RA-FLSs compared with three control RA-FLSs.

Proteins	Fold change
Tumor necrosis factor α converting enzyme	5.53
Matrix metalloproteinase 2	5.05
Platelet derived growth factor subunit AA	4.96
A disintegrin and metalloproteinase 9	3.03
Fibroblast growth factor 6	0.29
Interleukin-1 receptor antagonist	0.28
Transferrin	0.28
Fibroblast growth factor 4	0.26
Bone morphogenetic protein 9	0.25
Interferon-γ-inducible protein 10	0.23
Ectodysplasin A2 receptor	0.20
Albumin	0.15
C-C motif chemokine ligand 16	0.14

### Circ_0088194 Sponges miR-766-3p in RA-FLSs

**Figure 4:**
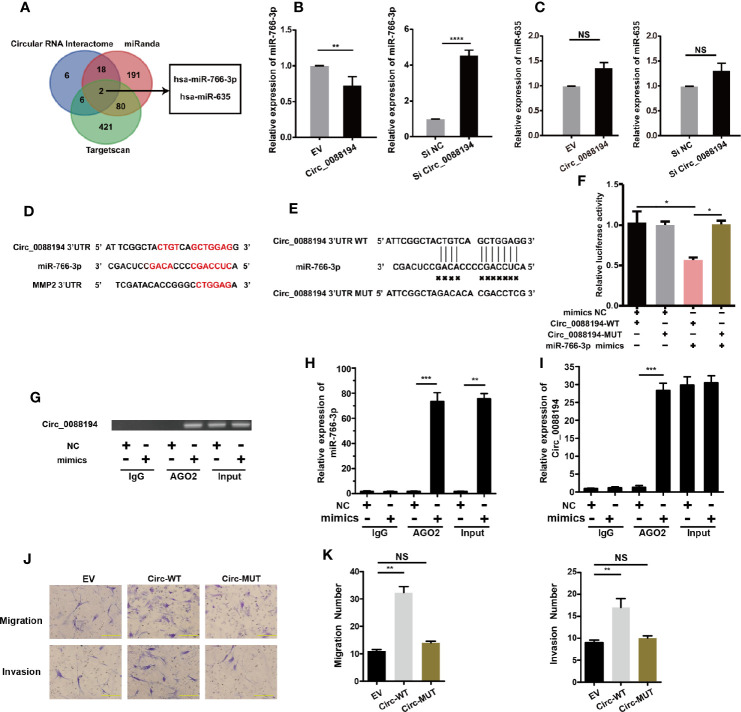
Circ_0088194 act as sponge of miR-766-3p in RA-FLSs. **(A)** Schematic illustration showing the identification of two miRNAs, hsa-miR-766-3p and hsa-miR-635, predicted from three available databases (miRanda, circular RNA interactome, and Targetscan). **(B, C)** QRT-PCR determination of the expression of miR-766-3p **(B)** and miR-635 **(C)** in RA-FLSs. The cells were transfected with adenovirus expressing Circ_0088194 or Circ_0088194#1 siRNA. **(D)** The miR-766-3p MREs in the sequences of Circ_0088194 and *MMP2* as identified using bioinformatic analysis. **(E)** Wild-type (WT) or mutant (MUT) miR-766-3p target sequences of Circ_0088194. **(F)** The miR-766-3p mimics reduced the luciferase activities of WT not MUT Circ_0088194 reporter genes, as assessed using dual-luciferase reporter assays. **(G)** RA-FLSs were transfected with the miR-766-3p mimics or the negative control. Anti-AGO2 RIP was used to investigate AGO2 binding to Circ_0088194 and miR-766-3p; IgG was used as a negative control. **(H, I)** The expression levels of Circ_0088194 and miR-766-3p, as detected using qRT-PCR. **(J, K)** The migratory and invasive activities of RA-FLSs were evaluated by Transwell migration and Matrigel invasion assays. RA-FLSs were transfected with the adenovirus encoding Circ_0088194 or the adenovirus encoding Circ_0088194 with mutated miR-766-3p binding sites. Scale bar, 500 µm. Data are shown as mean ± SD. NS, not significant. *indicates *p* < 0.05, **indicates *p <* 0.01, ***indicates *p <* 0.001, and ****indicates *p <* 0.0001.

It has been reported that CircRNAs can regulate gene expression *via* their miRNA responsive elements (MREs), which function as miRNA sponges in the cytoplasm (32). Circ_0088194 is localized mostly in the cytoplasm of RA-FLSs and bioinformatic analysis using three publicly available databases (TargetScan, miRanda, and circular RNA interactome) indicated that miR-766-3p and miR-635 might bind to Circ_0088194 and the 3′-untranslated region (UTR) of *MMP2* mRNA (**Figure 4A**). This led us to hypothesize that Circ_0088194 functions as a miRNA sponge to promote MMP2 expression in RA-FLSs. Firstly, we found that miR-766-3p expression increased in RA-FLSs after Circ_0088194 knockdown, while miR-766-3p expression decreased when Circ_0088194 was overexpressed (**Figure 4B**). However, the expression of miR-635 did not change after Circ_0088194 overexpression or knockdown (**Figure 4C**). Furthermore, we performed dual-luciferase assays to confirm the binding between Circ_0088194 and miR-766-3p (**Figures 4D, E**). The results showed that miR-766-3p mimics decreased the luciferase activity of the wild-type Circ_0088194 vector, but not that of the mutant Circ_0088194 vector (**Figure 4F**). A previous study indicated that miRNAs bind to MREs in an Argonaute 2 (AGO2)-dependent manner (33). Thus, we performed an RNA immunoprecipitation (RIP) experiment using anti-AGO2 antibodies to confirm the direct binding between miR-766-3p and Circ_0088194. Indeed, Circ_0088194 and miR-766-3p were enriched in the AGO2 immunoprecipitates relative to the IgG immunoprecipitates (**Figures 4G, I**). Importantly, we observed that overexpression of Circ_0088194 with mutated miR-766-3p binding sites had no significant effect on the migration and invasion of RA-FLSs (**Figures 4J, K**). Collectively, our findings suggested that Circ_0088194 acts as a sponge for miR-766-3p.

### MiR-766-3p Inhibits the Invasion and Migration of RA-FLSs

**Figure 5:**
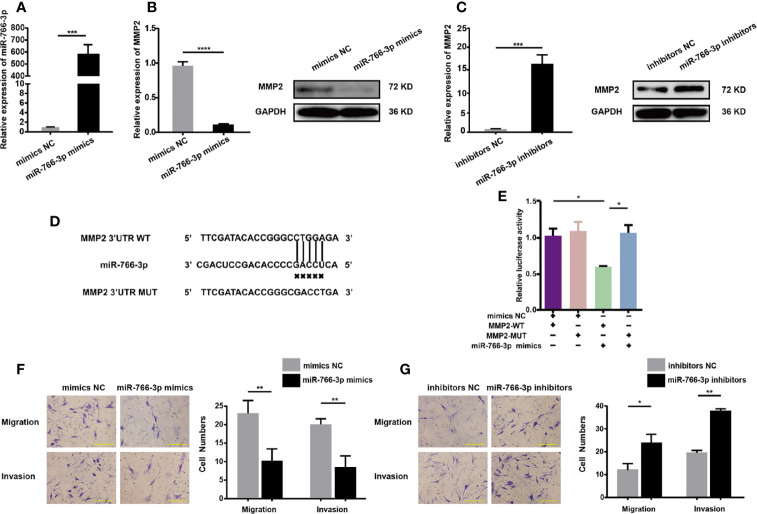
MiR-766-3p inhibits RA-FLS migration and invasion. **(A)** RA-FLSs were transfected with the miR-766-3p mimics or the negative control (NC) at a final concentration of 50 nM. The expression level of miR-766-3p was determined using qRT-PCR. **(B)** QRT-PCR and Western blotting determination of *MMP2* expression in RA-FLSs. RA-FLSs were transfected with miR-766-3p mimics. **(C)** QRT-PCR and Western blotting detection of MMP2 expression in RA-FLSs. RA-FLSs were transfected with miR-766-3p inhibitors. **(D)** Wild-type (WT) or mutant (MUT) miR-766-3p target sequences in the *MMP2* 3′ UTR. **(E)** The miR-766-3p mimics reduced the luciferase activities of WT but not MUT *MMP2* reporter gene, as detected using dual-luciferase reporter assays. **(F)** Assays of RA-FLSs migration and invasion in cells transfected with miR-766-3p mimics. Scale bar, 500 µm. **(G)** Assays of RA-FLSs migration and invasion in cells transfected with miR-766-3p inhibitors. Scale bar, 500 µm. Data are shown as mean ± SD. *indicates *p* < 0.05, **indicates *p <* 0.01, ***indicates *p <* 0.001, and ****indicates *p <* 0.0001.

The impact of miR-766-3p mimics on *MMP2* expression and RA-FLS migration and invasion was studied by transfecting RA-FLSs cells with miR-766-3p mimics or inhibitors, respectively. Transfection of miR-766-3p mimics (**Figure 5A**) reduced the expression of *MMP2* mRNA and protein in RA-FLSs (**Figure 5B**). Conversely, transfection of miR-766-3p inhibitors increased the expression of *MMP2* mRNA and protein (**Figure 5C**). Next, we constructed luciferase reporter plasmids containing the WT 3′-UTR of the *MMP2* mRNA and a version of the 3′-UTR of the *MMP2* mRNA in which the miR-766-3p binding sites were mutated (**Figure 5D**). The results demonstrated that in the presence of the miR-766-3p mimics, the luciferase activity from the WT construct decreased. However, the mimics had any effect on the luciferase activity from the MUT construct (**Figure 5E**). These results indicated that *MMP2* is a target of miR-766-3p. Importantly, Transwell assays revealed that overexpression of miR-766-3p reduced RA-FLS migration and invasion (**Figure 5F**); while the miR-766-3p inhibitors increased RA-FLS migration and invasion (**Figure 5G**). Thus miR-766-3p was confirmed to reduce *MMP2* expression and inhibit the migration and invasion of RA-FLSs.

### Circ_0088194 Promotes RA-FLSs Migration and Invasion *via* the miR-766-3p/MMP2 Axis

**Figure 6:**
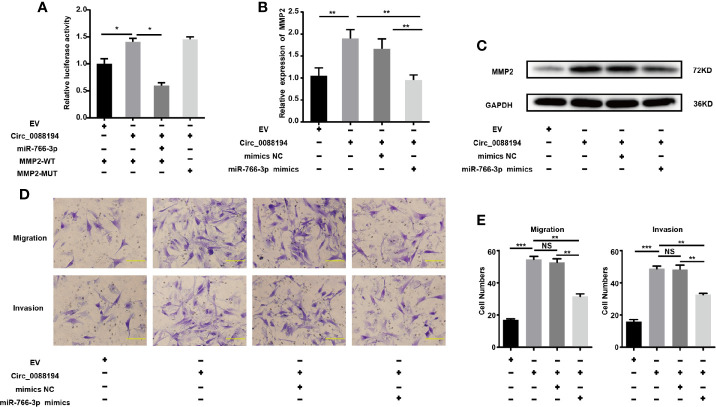
Circ_0088194 promotes RA-FLSs migration and invasion *via* miR-766-3p/MMP2 axis. **(A)** The increased luciferase activity of WT-MMP2 treated with Circ_0088194 adenovirus was blocked by co-transfection with miR-766-3p mimics. **(B)** In RA-FLSs, the *MMP2* mRNA levels were determined using qRT-PCR. MiR-766-3p mimics and Circ_0088194 adenovirus were co-transfected into RA-FLSs. **(C)** Western blotting analysis of MMP2 protein levels in RA-FLSs. RA-FLSs were co-transfected with miR-766-3p mimics and Circ_0088194 adenovirus. **(D, E)** The migratory and invasive ability of RA-FLSs. RA-FLSs were co-transfected with miR-766-3p mimics and Circ_0088194 adenovirus. Scale bar, 500 µm. Data are shown as mean ± SD. NS, not significant. *indicates *p* < 0.05, **indicates *p <* 0.01, and ***indicates *p <* 0.001.

To test whether Circ_0088194 promotes *MMP2* expression by sponging miR-766-3p, we performed dual-luciferase reporter assays. The result showed that the miR-766-3p mimics reduced the luciferase activities from the reporter plasmid containing the potential binding sequence of Circ_0088194-WT or MMP2-WT (**Figure 4F**, **5E**). However, luciferase activities were not affected when mutant Circ_0088194-MUT and MMP2-MUT were used. Circ_0088194 overexpression increased the luciferase activity of MMP2-WT relative to the corresponding MMP2-MUT. Interestingly, when the miR-766-3p mimics were co-transfected, the increased luciferase activity from MMP2-WT in response to Circ_0088194 overexpression was abrogated (**Figure 6A**). MiR-766-3p mimic co-transfection blocked the upregulation of the *MMP2* mRNA and protein levels induced by Circ_0088194 overexpression in RA-FLSs (**Figures 6B, C**). Consistently, transfection of Circ_0088194 promoted RA-FLSs migration and invasion, which could be blocked by co-transfection with miR-766-3p mimics (**Figures 6D, E**). Taken together, these results indicated that Circ_0088194 regulates the expression of *MMP2* by sponging miR-766-3p, thereby promoting migration and invasion.

### The m^6^A Modification of Circ_0088194 Exists in RA-FLSs

**Figure 7:**
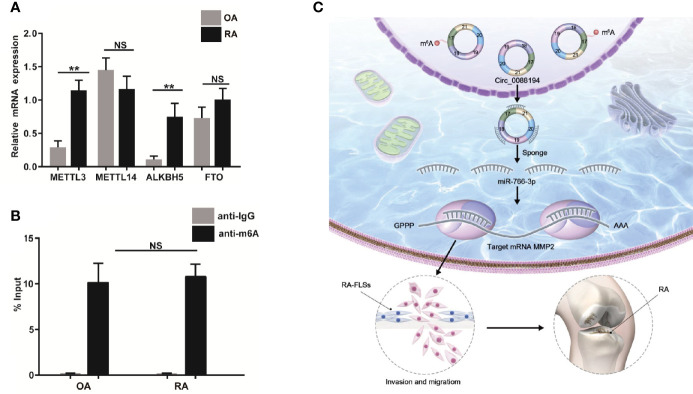
Circ_0088194 m^6^A modification in RA-FLSs. **(A)** QRT-PCR determined the mRNA level of four components of the m^6^A methylase complex methyltransferase-like 3 (METTL3), alkB homolog 5 (ALKBH5), methyltransferase-like 14 (METTL14), and fat mass and obesity associated genes (FTO) in RA-FLSs and OA-FLSs. **(B)** Anti-m^6^A antibody-enriched m^6^A-modified total RNA immunoprecipitation followed by qRT-PCR determined the m^6^A-modified Circ_0088194 levels in RA-FLSs and OA-FLSs. The negative control for immunoprecipitation comprised rabbit IgG. The percentages of input are shown. **(C)** A working model of the Circ_0088194/miR-766-3p/MMP2 signaling axis in the promotion of the migration and invasion of RA-FLSs and RA pathogenesis. Data are shown as mean ± SD. NS: not significant. **indicates *p <* 0.01.

The m^6^A modification occurs in circRNAs as a mechanism to drive translation initiation (34, 35). Using the SRAMP website (http://www.cuilab.cn/sramp), we predicted the m^6^A modification sites on Circ_0088194. Next, we verified the mRNA levels of four essential components of the m^6^A methylase complex: alkB homolog 5 (ALKBH5), methyltransferase-like 14 (METTL14), fat mass and obesity associated protein (FTO), and methyltransferase-like 3 (METTL3), in RA-FLSs and OA-FLSs using qRT-PCR. The mRNA levels of *METTL3* and *ALKBH5* in RA-FLSs were markedly higher than those in OA-FLSs (**Figure 7A**). However, the *METTL14* and *FTO* expression levels did not differ between OA-FLSs and RA-FLSs. To test whether the m^6^A modification of Circ_0088194 differed in RA-FLSs compared with that in OA-FLSs control cells, we performed methylated RNA immunoprecipitation using the rabbit anti-m^6^A polyclonal antibody to enrich m^6^A-modified total RNA, followed by qRT-PCR of Circ_0088194. We found that the relative contents of m^6^A-modified Circ_0088194 did not differ between RA-FLSs and OA-FLSs (**Figure 7B**). These results suggested a negligible effect of this modification on Circ_0088194’s activity in miR-766-3p binding and *MMP2* expression.

## Discussion

Recently, thousands of circRNAs have been identified from mammalian transcriptomes, and dysregulation of circRNAs is involved in the pathogenesis of various diseases, including RA (36–38). However, the roles of circRNAs in RA-FLSs are largely unknown. In this study, we identified a novel circRNA, Circ_0088194, which was upregulated in RA-FLSs and correlated positively with the RA disease activity score in 28 joints. Overexpression of Circ_0088194 promoted the migration and invasion of RA-FLSs. Mechanistically, Circ_0088194 acted as a miRNA sponge to reduce the level of miR-766-3p, thereby relieving miR-766-3p’s repressive effect on its target, MMP2, ultimately promoting migration and invasion. These results indicated that the Circ_0088194/miR-766-3p/MMP2 axis has an important function in regulating RA-FLS migration and invasion (**Figure 7C**).

Invasion and migration of RA-FLSs leads to cartilage degradation and subsequent bone destruction, which cause a high level of disability in patients with RA. Therefore, it is imperative to identify invasion-related factors and determine the molecular mechanisms of RA-FLS invasion and migration. Herein, molecular experiments showed that overexpression of Circ_0088194 promoted migration and invasion of RA-FLSs, while downregulation of Circ_0088194 had the reverse effect. Importantly, in RA, Circ_0088194 levels correlated with the disease activity score in 28 joints. These data implied that in RA-FLSs, Circ_0088194 might contribute to increased rheumatoid synovial aggression and hyperplasia, ultimately leading to joint destruction.

CircRNAs may play specific roles in cellular physiology, and several possible functions have been proposed, including miRNA sponges (33), protein binding (39), and regulation of translation and translation into proteins (40). When acting as miRNA sponges, MREs in circRNAs reduce the levels of endogenous miRNAs capable of binding to their target mRNAs, thereby increasing mRNA translation, which is the most important and frequently reported function of circRNAs. For example, circRNA ciRS-7, which contains 74 binding sites for miR-7, was the first circRNA to be established as a miRNA sponge and has been reported to participate in the development of many cancers (41). In this study, we found that Circ_0088194 promoted RA-FLSs migration and invasion by increasing the expression of invasion-related gene, *MMP2.* Bioinformatic analyses demonstrated that *MMP2* and Circ_0088194 share an MRE for miR-766-3p. Therefore, we hypothesized that Circ_0088194 promotes RA-FLS migration and invasion *via* the miR-766-3p/MMP2 axis. To verify our hypothesis, luciferase reporter assays confirmed the direct interaction between Circ_0088194 and miR-766-3p, and between miR-766-3p and *MMP2*. Importantly, miR-766-3p reverses the ability of Circ_0088194 to upregulate *MMP2* expression and promote RA-FLS migration and invasion. Thus, we demonstrated that Circ_0088194 acts as a sponge toward miR-766-3p to promote RA-FLSs migration and invasion by upregulating *MMP2* expression at the post-transcription level. This consists with previous studies which showed knockdown of MMP2 reduced the invasion and migration of RA-FLSs (30, 42, 43).Moreover, MMP (including MMP-2) is considered as the primary therapy for RA (44, 45). However, the other study indicated that MMP-2 suppresses RA synovial fibroblast-mediated cartilage degradation; MMP-2 KO mice developed arthritis of greater clinical and histological severity than wild-type mice (46). Therefore, the role of MMP2 in the progression of RA-FLS is still controversial. We speculate that the varied roles of MMP2 in the progression of RA may be associated with the inconsistency of microenvironment and experimental conditions. Additionally, we cannot exclude the possibility that other pathways might also be involved Circ_0088194’s effects in addition to the miR-766-3p/MMP2 axis, which might also play important roles in RA-FLSs progression. These the other potential functions of Circ_0088194 beyond acting as a miRNAs sponge in RA-FLSs merit further exploration.

A recent study showed that the m^6^A modification on circRNAs mediate their stability by increasing circRNA association with YTH N6-methyladenosine RNA binding protein 2 (YTHDF2) in a heat-responsive protein 12 (HRSP12)-dependent manner (20). We found that upregulation of Circ_0088194 correlated with elevated RA-FLSs invasion and migration. Therefore, we suspected that the m^6^A modification might affect the stability of Circ_0088194 and account for Cir_0088194 differences between RA-FLSs and OA-FLSs. The prediction website showed there are three methylation sites with high confidence on Circ_0088194. As expected, our study showed that the mRNA levels of two components from the m^6^A methylase complex, METTL3 and ALKBH5, were significantly higher in RA-FLSs than in OA-FLSs. To our surprise, such differences did not affect the total amount of m^6^A-modified Circ_0088194. These observations suggested that the elevated levels of Circ_0088194 in RA-FLSs compared with those in OA-FLSs might have resulted from different mechanisms to control Circ_0088194 production, such as Circ_0088194 alternative splicing, a hypothesis that requires further exploration.

To the best of our knowledge, this is the first report that Circ_0088194 acts as a pathogenic element in the progression of human RA by regulating the expression of collagenase MMP2 and promoting RA-FLS migration and invasion. However, we did not test this hypothesis in experimental RA models, and additional studies with large sample size and patients of different ethnicities and regions might be required. Our findings identified the Circ_0088194/miR-766-3p/MMP2 pathway as a novel therapeutic target to prevent and treat human RA. The significant positive correlation between the RA disease score and the RA-FLS Circ_0088194 contents supported our hypothesis.

## Data Availability Statement

The raw data supporting the conclusions of this article will be made available by the authors, without undue reservation.

## Ethics Statement

The ethics committee of Southern Medical University approved the study and associated protocols (Guangzhou, China, NFEC-20120201). The patients/participants provided their written informed consent to participate in this study. Written informed consent was obtained from the individual(s) for the publication of any potentially identifiable images or data included in this article.

## Author Contributions

YC, QO, RL, SX, and DZ performed the molecular genetic analyses. MY, QO, and QH participated in experimental design and statistical analyses. YC drafted the manuscript, and G-PS edited the manuscript. All authors contributed to the article and approved the submitted version.

## Funding

This study was supported by the National Natural Science Fund of China (no. 81771747), Guangdong Provincial Natural Science Fund (No. 2017A030313475), and Presidential Foundation of the Nanfang Hospital (No. 2019C008).

## Conflict of Interest

The authors declare that the research was conducted in the absence of any commercial or financial relationships that could be construed as a potential conflict of interest.
