# Supplementary material for: Circ_0088194 Promotes the Invasion and Migration of Rheumatoid Arthritis Fibroblast-Like Synoviocytes via the miR-766-3p/MMP2 Axis
Source: Front Immunol. 2021 Feb 22;12:628654. doi: 10.3389/fimmu.2021.628654 (PMC7937802; doi:10.3389/fimmu.2021.628654)
Supplement: Supplementary file 1 [file DataSheet_1.doc]

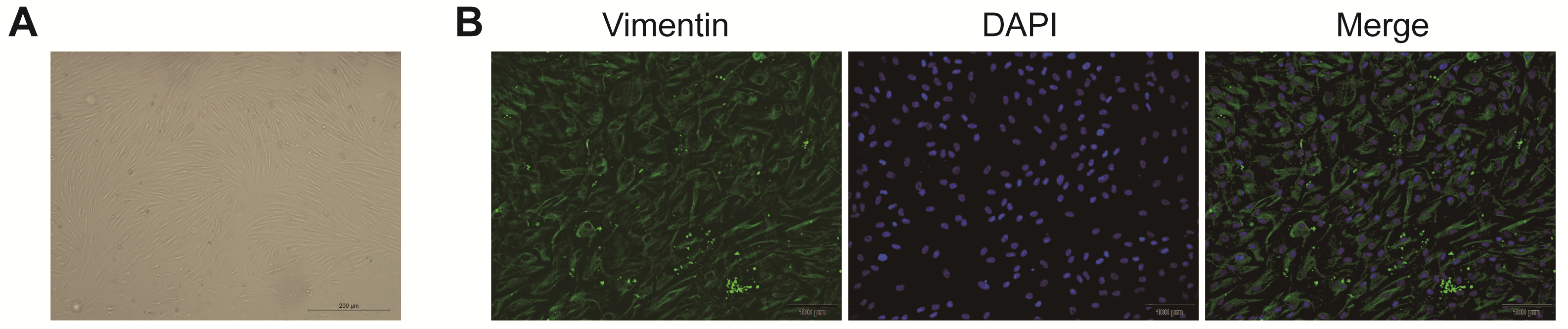


**Supplementary Figure 1** Culture and identification of fibroblast-like synoviocytes (FLSs)

1. Primary cultured FLSs on 30 day. Scale bar, 200µm. (B) Identification of FLSs. To observe the expression of vimentin in FLSs by immunofluorescence. Scale bar, 100µm.

**A**


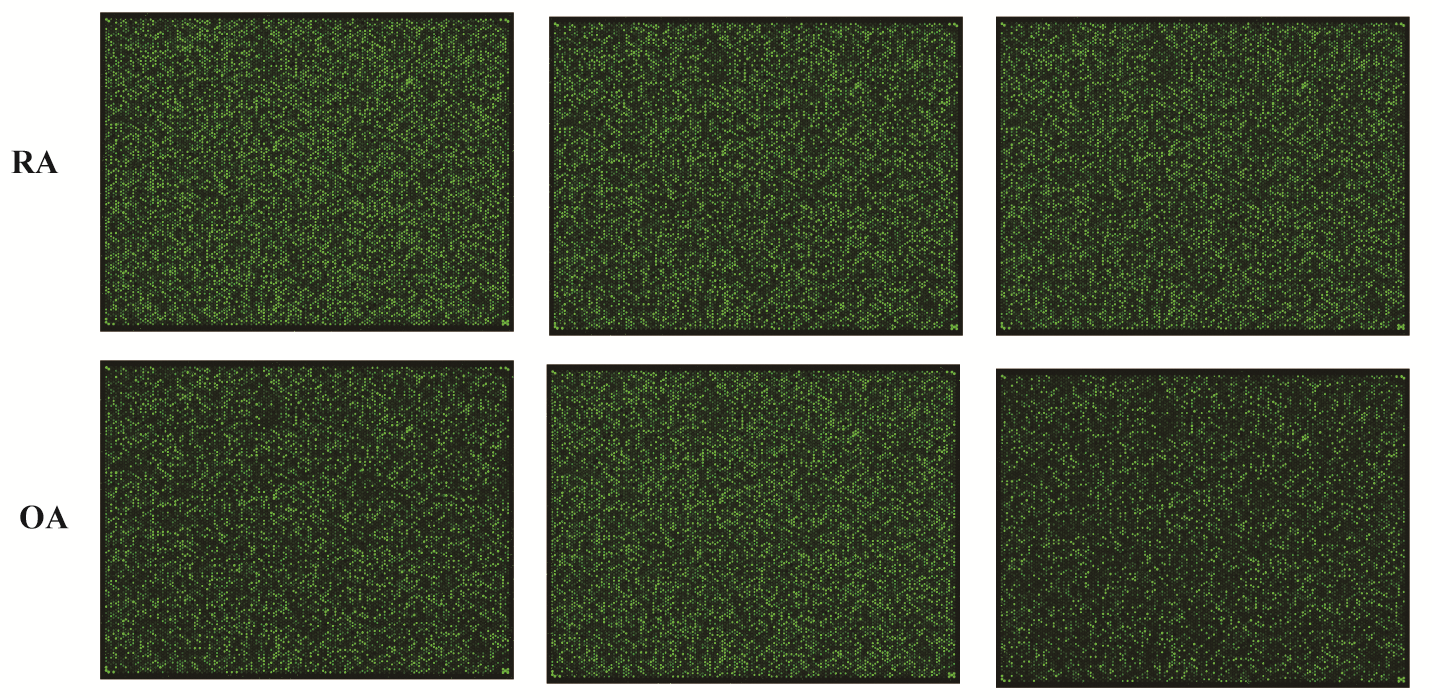


**Supplementary Figure 2** Raw graphs of circular RNAs microarray. (A) Three cell samples in the top row are rheumatoid arthritis fibroblast-like synoviocytes (RA- FLSs), three on the bottom row are osteoarthritis fibroblast-like synoviocytes (OA-FLSs).

**Supplementary Table S1.** Primers and sequences used in this study.

| **Name** | **Sequence** |
| --- | --- |
| Hsa_circ_0088194 | F:5'GCTCAACCATCACTGCCAA3'  R:5'CCAGTGGCTCAGACTGCTTT3' |
| Hsa_circ_0088200 | F:5'TGCTGAACGAACTGCCCATA3'  R:5'ATTTCAGGTTCTTTGGCTGTCG3' |
| Hsa_circ_0012103 | F:5'TGCTGAATTAGCCACCAAGTAC3'  R:5'AGCGGAGTCAAAGGAAAGAAC3' |
| Hsa_circ_0034953 | F:5'AGCCTTATCGCCAAGCACTG3'  R:5'AAAGGGAAGTCCTGCGATACC3' |
| TNC mRNA | F:5'CAACCATCACTGCCAAGTTCACAAC3'  R:5'CGCCTCAGCCTTATCACCATTCAG3' |
| MMP2 mRNA | F:5′CACCTACACCAAGAACTTCC3′  R:5′AACACAGCCTTCTCCTCCTG3′ |
| GAPDH | F:5'GGGAAACTGTGGCGTGAT3'  R:5'GAGTGGGTGTCGCTGTTGA3' |
| Si-hsa_circ_0088194_001 | CCAAGTTCACAACAGAAGC |
| Si-hsa_circ_0088194_002 | TCACAACAGAAGCCGAACC |
| Si-hsa_circ_0088194_003  Si-MMP2 | GTTCACAACAGAAGCCGAA  AGUUGGCAGUGCAAUACCUGA |
| Si NC | RiboBio |
| MiR-766-3p | F:5'TGAGAACCACGTCTGCTCTGAG3'  R: Universal Reverse Primer |
| MiR-635 | F:5'ACTTGGGCACTGAAACAATGT3'  R: Universal Reverse Primer |
| U6 | F:5'GGAACGATACAGAGAAGATTAGC3'  R:5'TGGAACGCTTCACGAATTTGCG3' |
| MiR-766-3p mimics | F:5'ACUCCAGCCCCACAGCCUCAGC3'  R:5'GCUGAGGCUGUGGGGCUGGAGU3' |
| MiR-766-3p inhibitors | GCUGAGGCUGUGGGGCUGGAGU |
| Mimics NC | RiboBio |
| Inhibitors NC | RiboBio |
| Biotin-labeled Circ_0088194 | CACAGTGGAGTATGCTCTGACCGACCTCGAGCCTGCCACGGAATACACACTGAGAATCTTTGCAGAGAAAGGGCCCCAGAAGAGCTCAACCATCACTGCCAAGTTCACAACAGAAGCCGAACCGGAAGTTGACAACCTTCTGGTTTCAGATGCCACCCCAGACGGTTTCCGTCTGTCCTGGACAGCTGATGAAGG |
